# The cognitive profile of type 1 Gaucher disease patients

**DOI:** 10.1007/s10545-012-9460-7

**Published:** 2012-02-21

**Authors:** Marieke Biegstraaten, Keith A. Wesnes, Cécile Luzy, Milan Petakov, Mirando Mrsic, Claus Niederau, Pilar Giraldo, Derralynn Hughes, Atul Mehta, Karl-Eugen Mengel, Carla E. M Hollak, László Maródi, Ivo N. van Schaik

**Affiliations:** 1 Department of Internal Medicine, Academic Medical Center, F5-169, Box 22700, 1100 DE Amsterdam, The Netherlands; 2 United BioSource Corporation, Goring-on-Thames, UK; 3 Actelion Pharmaceuticals Ltd, Allschwil, Switzerland; 4 Institute of Endocrinology, Clinical Center of Serbia, Belgrade, Serbia; 5 Department of Hematology, University Hospital Center, Zagreb, Croatia; 6 Katholische Kliniken Oberhausen, Academic Teaching Hospital of the University of Duisburg-Essen, Oberhausen, Germany; 7 CIBERER (Centro de Investigación Biomédica en Red de Enfermedades Raras), Zaragoza, Spain; 8 Royal Free and University College Medical School, London, UK; 9 Universitaets Kinderklinik, Mainz, Germany; 10 University of Debrecen, Debrecen, Hungary; 11 Department of Neurology, Academic Medical Center, H2-227, Box 22700, 1100 DE Amsterdam, The Netherlands

## Abstract

**Background:**

The absence of neurological symptoms and signs is traditionally considered mandatory for a diagnosis of type 1 Gaucher disease (GD1), but in recent years many reports have emerged on neurological manifestations in GD1 patients. Nevertheless, it has been unclear whether cognitive deficits are part of the disease as well.

**Methods:**

Cognitive function was assessed in a large cohort of GD1 patients with the use of the CDR system, a set of computerised cognitive tests. Testing was performed at baseline and every 6 months thereafter during a two-year study period.

**Results:**

Our patient cohort (84 patients, median age 40 years, median time from diagnosis 15 years) showed mild deficits relative to healthy age-matched subjects on the composite scores: power of attention (Z-score (mean ± SD) -0.9 ± 1.37) and speed of memory (Z-score (mean ± SD) -1.39 ± 1.49). No decline in cognitive function was seen during the two-year period. Age correlated with the composite scores variability of attention and quality of working memory. Moreover, severely affected patients (Zimran severity score (SSI) ≥ 15) scored more poorly compared to mildly affected patients (SSI ≤ 5) on the composite measure power of attention, reflecting the ability to concentrate.

**Conclusions:**

GD1 patients exhibit mild deficits in power of attention and speed of memory, reflecting a decreased ability to focus attention and process information, together with a slowing in the speed of retrieval of items from memory. The clinical relevance of these findings is uncertain.

## Background

Gaucher disease is one of the most common lysosomal storage diseases with an estimated prevalence of 1 in 40,000 to 50,000 live births (Grabowski 2008). The disease is caused by mutations in the glucocerebrosidase (GBA) gene resulting in a decreased activity of the enzyme and subsequent accumulation of glucocerebrosides in macrophages throughout the body. The absence of nervous system involvement is used as a criterion in the classic definition of type 1 Gaucher disease (GD1). However, peripheral as well as central nervous system disease has been described in type 1 Gaucher disease. We recently reported an increased prevalence and incidence of polyneuropathy in GD1 patients compared with the general population (Biegstraaten et al 2010). In addition, GD1 and GBA mutations have been associated with Parkinson disease (PD) and Dementia with Lewy Bodies (DLB) (Goker-Alpan et al 2006; Sidransky et al 2009). Moreover, an autopsy study on 14 Gaucher brains showed neuropathological changes not only in type 2 and 3 patients but also in patients with type 1 disease. Selective vulnerability of the hippocampal CA2-4 regions, cerebral cortical layers 3 and 5 and calcarine cortex layer 4b with normal adjacent regions have been found in all three types of Gaucher disease, although there were qualitative and quantitative differences between the three types; neurodegeneration predominated in type 2 and 3 disease, whereas astrogliosis was the only manifestation in type 1 patients (Wong et al 2004).

Following the observation of a case of dementia during a clinical trial with the substrate reduction therapy miglustat (Zavesca^TM^, Actelion Pharmaceuticals Ltd, Allschwil, Switzerland) (Elstein et al 2005), the question arose whether the cognitive decline in this patient was miglustat-related, a coincidence or part of GD1. As part of a post-marketing surveillance commitment to the European Medicines Agency (EMA) in relation to the registration of miglustat, we conducted a multi-centre study, under the auspices of the European Working Group on Gaucher Disease, to investigate comorbidities in GD1, with a special focus on peripheral neuropathy; the main findings have been reported elsewhere (Biegstraaten et al 2010). Besides, the cognitive profile of GD1 patients was investigated. In this paper we present data on the cognitive profile of GD1 patients and the changes over a two-year study period.

## Methods

### Study design

This 2-year prospective, longitudinal, observational cohort study was conducted in eight centres across seven countries in Europe.

### Patients

Eligible adult patients diagnosed with GD1 by glucocerebrosidase assay or molecular genetic analysis, attending routine clinical visits, and receiving either no treatment or enzyme replacement therapy (ERT) were enrolled between 3 May 2005 and 28 December 2006. Those with a history of manifestations or mutations associated with type 3 disease were excluded. Patients were also excluded if they were undergoing or had undergone treatment with miglustat or an investigational agent, or if they had neurological or psychiatric conditions that could influence cognitive performance. Finally, patients with a yet undefined mutation were excluded from the analyses.

Patients who attended at least one post baseline visit were included in the analyses. All patients provided written, informed consent before participation. The study protocol was approved by independent local ethics committees, and was conducted in accordance with the Declaration of Helsinki, 1964, and subsequent revisions.

### Assessments

Disease severity was assessed at baseline using the Zimran severity score index (SSI) (Zimran et al 1992). On study entry (baseline), and every six months thereafter, patients underwent cognitive function testing with the CDR system, a validated computerised cognitive assessment system, (Simpson et al 1991). The 30-minute test battery was administered in a quiet room, one-to-one, by trained administrators using standardised task instructions in the patient’s native language. The CDR system has been validated in the various languages required for the present study. Patients had two training sessions on the tests during the 14 days prior to baseline. During each test session, the following tests were administered: simple reaction time, digit vigilance, choice reaction time, spatial working memory, numeric working memory, delayed word recognition, delayed picture recognition and morse tapping (see (Wesnes et al 2000) for full task descriptions).

In addition to the analysis of individual task measures, six predefined, validated CDR composite scores were used: power of attention, continuity of attention, quality of episodic memory, quality of working memory, speed of memory and variability of attention (Table 1) (Wesnes et al 2000).

**Table 1:** CDR composite scores

Composite score	Measures contributing	Aspects of cognitive function involved
Power of attention	Simple reaction time	Ability to focus attention and ignore distraction. A measure of early information processing.
	Choice reaction time	
	Digit vigilance speed of detections	
Continuity of attention	Choice reaction time accuracy (%)	Ability to sustain attention (concentration, vigilance)
	Digit vigilance task detections (%)	
	Digit vigilance task – false alarms	
Quality of working memory	Numeric working memory & spatial working memory sensitivity scores	Ability to successfully hold information temporarily in both articulatory working memory and spatial working memory. Short-term memory.
Quality of episodic memory	Percentage overall accuracy on word and picture recognition tasks	Ability to encode, store and subsequently successfully retrieve verbal and non-verbal information. Long-term memory.
Speed of memory	Speeds of correct identifications in working memory, word recognition and picture recognition tasks	A measure of the time taken to process verbal and non-verbal information and to successfully retrieve the information from both working memory and episodic memory
Variability of attention	The coefficients of variance for simple reaction time, choice reaction time and digit vigilance speed of detections	A measure that reflects fluctuations in attention

### Analysis

To compare the cognitive profile of the GD1 population with the general population, age-matched scores were identified from the normative database of the CDR system in various age-bands (see Table 2). The normative database comprises data from over 5000 healthy individuals aged 18 to 87 years, who were free of any psychiatric or major medical condition at the time of testing (Wesnes 2006). The nationalities of the individuals were American, Belgian, Danish, Dutch, English, French, German, Swedish and Swiss, and around 2% of the individuals were Afro-Carribean or Asian, the rest being Caucasian.

**Table 2:** Patient characteristics

	All patients (*N* = 84)
**Demographics**	
Gender, male / female, *n* (%)	41 (49) / 43 (51)
Age in years, median (range)	40 (18-75)
Age-bands, years	
18-32, *n*	23
33-41, *n*	23
42-52, *n*	19
53-75, *n*	19
Country of origin	
Netherlands	21 (25%)
Germany	19 (23%)
Hungary	18 (21%)
Spain	6 (7%)
United Kingdom	3 (4%)
Croatia	4 (5%)
Serbia	13 (15%)
**GD1 characteristics**	
Genotype, *n* (%)	
N370S/N370S	11 (13.1%)
N370S/L444P	18 (21.4%)
N370S/84GG	2 (2.4%)
N370S/IVS2 + 1	1 (1.2%)
N370S/other	39 (46.4%)
N370S/unknown	8 (9.5%)
L444P/G377S	1 (1.2%)
RECNCI1/D140H	1 (1.2%)
R463C/R463C	1 (1.2%)
A446P/A446P	1 (1.2%)
R48Q/T323I	1 (1.2%)
Time from diagnosis in years, median (range)	15 (0-56)
Splenectomised, n (%)	26 (31)
SSI, median (range)	8 (2-21)
Plasma chitotriosidase^‡^ in nmol/ml.hour, median (range)	5,332 (100-49,860)
**ERT**	
Receiving ERT, *n* (%)	71 (84.5)
Duration in years, median (range)	2.1 (0.1-13.6)
Dosage in IU / kg / month, median (range)	54 (12-137)

^‡^data available from 82 patients (normal range, 4–120 nmol/ml.hour); ERT, enzyme replacement therapy.

Using the normative data, the patients’ data were Z-transformed and mean ± SD GD1 population Z-scores were calculated by age-group. Z-scores were transformed so that higher scores always indicate better performance. Furthermore, 95% confidence intervals (95%CIs) were calculated. In addition, repeated measures analysis of covariance (ANCOVA) was performed on the change from baseline data over the follow-up assessments (6, 12, 18 and 24 months), with each patient’s baseline value used as a covariate in the analysis.

Additional analyses were conducted to determine whether age, disease severity, treatment with ERT and/or genotype were associated with cognitive performance. In previous work, various cut off values on the SSI have been used to separate mild disease from severe disease. Mild disease has been defined by a SSI of ≤ 5, and severe disease by a score of ≥ 15 (Shitrit et al 2003), but a cut off value of 11 has also been used (Aker et al 1993). Consequently, subgroup analysis for both cut off values was undertaken. The following genotype subgroups were analysed: patients who were homozygous for N370S, patients who had at least one N370S allele, and patients who had at least one L444P mutation. Differences were tested with the Student’s t-test or Mann Whitney test, and Pearson’s correlation was used to assess correlations between variables.

As indicated before, the prevalence and incidence of polyneuropathy were investigated in the same study group. To assess whether the peripheral and central nervous system symptoms were related, multivariate analyses were performed using ANCOVA, fitting age as a covariate and disease severity and polyneuropathy as factors. Subsequently, ANCOVA was used correcting for baseline values to assess whether impairments changed differentially over time in the disease severity and polyneuropathy subgroups.

Data were collected by Actelion Pharmaceuticals Ltd, sponsor of the study. Statistical analyses were performed by co-author KAW, practice leader of United BioSource Corporation.

## Results

### Patients

From a total of 104 patients screened for inclusion in the original cohort, 102 patients attended at least one post baseline visit. One patient withdrew after screening due to an inability to commit to follow-up assessments, and one patient requested withdrawal after the first visit as she moved to another country. Of these 102 patients, 18 patients were excluded from the analyses for this study due to the use of sedative drugs (including opioid analgesics), concomitant neurological or psychiatric disorders, mutations associated with neuronopathic Gaucher disease or unknown mutations.

Of the remaining 84 patients, 78 completed the two years study. Four patients were withdrawn due to starting treatment with miglustat, one patient was non-compliant and one patient requested withdrawal from study for personal reasons. Patient characteristics are summarised in Table 2.

### Cognitive function tests

**Fig. 1:**
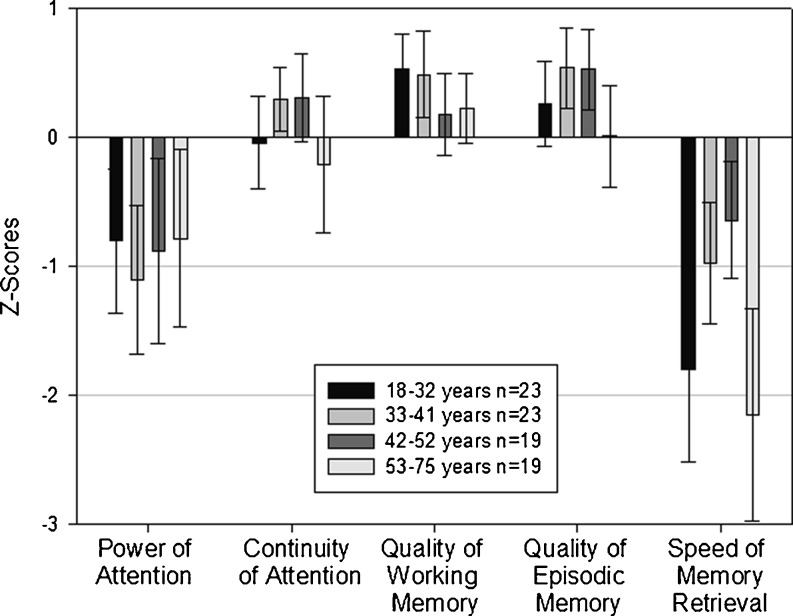
Z-scores at baseline by age quartiles, means with 95% confidence intervals

Taking all baseline results, patients with GD1 were mildly impaired relative to age-matched subjects on the composite scores power of attention and speed of memory (Fig. 1). For the whole population, power of attention showed a mean ± SD Z-score of -0.9 ± 1.37 and speed of memory showed a mean ± SD Z-score of -1.39 ± 1.49. The results were normally distributed which implies that the mean Z-scores were not due to a few outliers but rather reflecting mild deficits in the whole cohort. For power of attention 36 patients had deficits of 1 SD or greater and 16 had deficits of 1.96 or greater. For speed of memory 47 patients had deficits of 1 SD or greater and 24 had deficits of 1.96 or greater.

Since the GD1 patients showed decreased speed measures but scored the same or even slightly better in the accuracy scores compared to the normative population, an additional ANCOVA analysis was conducted on the speed measures, fitting the accuracy scores as covariates. This analysis showed that the deficits in power of attention and speed of memory were independent from the accuracy scores, indicating that the slowed speed scores reflected cognitive deficits and not differences in response strategy.

### Post baseline measurements

Power of attention showed a significant decline in performance of around -0.3 at each of the post baseline visits (p < 0.02), but no main effect of time-point was seen (F(3,83) = 0.63, p = 0.6), indicating that patients declined from baseline, but that there was no progressive decline thereafter. For continuity of attention, the data showed stable performance over time. Improvements in performance on quality of episodic memory from baseline to each of the subsequent assessments were found (p < 0.002), but there was no significant main effect of time point (F(3,82) = 0.84, p = 0.8). Performance on the speed of memory tasks was better at each of the post baseline time-points (p < 0.001), and a main effect of time-point was found (F(3,82) = 3.04, p = 0.03). The data of Quality of Working Memory and Variability of Attention showed fluctuations in performance over the duration of the study.

### Relation between cognitive function and age, disease severity, ERT, genotype and polyneuropathy

Pearson’s correlation showed correlations between age and variability of attention (r_p_ = -0.312, p < 0.005) and between age and quality of working memory (r_p_ = -0.232, p < 0.05): older patients scored worse on these scores than younger patients did. Besides, the SSI correlated with power of attention (r_p_ = -0.265, p < 0.02).

Cognitive function did not differ between ERT treated and untreated patients. Analyses by genotype revealed that patients carrying at least one N370S mutation (n = 79) did not differ to those without such a mutation (n = 5). Likewise, cognitive performance of N370S homozygotes and L444P heterozygotes did not differ from the results in patients with other genotypes.

Of the 84 patients included in the analyses, 14 (16.7%) were diagnosed with sensory motor axonal polyneuropathy (Biegstraaten et al 2010). Polyneuropathy patients were older than patients without polyneuropathy (60 versus 37 years, p < 0.001). The median SSI did not differ between patients with polyneuropathy and patients without polyneuropathy (8.5 versus 7, p = 0.154). There was no correlation between age and the SSI (r_p_ = 0.137, p = 0.219), nor did age differ between the disease severity groups. To evaluate the relationship of disease severity (using both criteria as discussed above) and polyneuropathy to the baseline scores of the patients, factorial ANCOVAs were performed, fitting the presence of polyneuropathy and the disease severity as factors, and age as a covariate. No effects of polyneuropathy were identified for power of attention, but severely affected patients (SSI ≥ 15) scored worse compared to mildly affected patients (SSI ≤ 5) on this composite measure (-1.93 versus -0.47, 95%CI -0.21, -2.72, p < 0.025). No differences were found if a cut-off value of 11 was used. There was no interaction between polyneuropathy and disease severity. No differences were found for the other measures. The cognitive profile did not change differentially over time in the disease severity and polyneuropathy subgroups.

## Discussion

This study on cognitive function in GD1 patients revealed mild deficits for power of attention and speed of memory in this patient group, reflecting a poorer ability to focus attention and a slowed retrieval of information held in memory in comparison to age-matched healthy controls. Unfortunately, subjective cognitive complaints were not assessed in this study. However, experience shows us that GD1 patients usually do not report difficulties in conducting daily activities or cognitive problems (personal communications). This suggests that the deficits in our patients were subtle and of doubtful clinical relevance. Although there was no necessity for polyneuropathy and cognitive dysfunction to coincide we decided to assess whether the neurological symptoms were related. The analyses revealed that these symptoms were unrelated, suggesting different underlying pathological mechanisms.

The cognitive profile of GD1 patients shows some similarities with the profile of type 3 patients: a study in GD3 children has shown that the cognitive deficits in these patients typically affect general nonverbal skills with a relative preservation of verbal skills. About 60% had below-average intellectual skills, and the weaknesses were specifically observed in the areas of processing speed, visual-spatial relationships, and perceptual organization skills (Goker-Alpan et al 2008). Thus, GD1 as well as GD3 patients exhibit decreased speed measures. However, type 3 patients have a broader spectrum of cognitive function deficits. Moreover, GD3 patients encounter problems in conducting their daily activities, while patients with GD1 do not. It is still unclear why some patients develop type 1 disease and others type 3 disease, despite the same underlying defective gene, and in some instances even the same underlying genotype.

It has been hypothesised that a toxic metabolite is responsible for the nervous system damage in type 2 and 3 patients; glucosylsphingosine, a glycosphingolipid that is also degraded by glucocerebrosidase, is a highly cytotoxic compound that has been demonstrated to be elevated in spleen and liver samples of Gaucher patients of all types, while levels in brain samples were elevated only in those with neuronopathic forms (Orvisky et al 2002). However, it is still unclear why these levels are only elevated in neuronopathic Gaucher disease patients. Moreover, this does not explain the mild cognitive deficits in type 1 patients as found in the present study.

A second hypothesis is that the (minimal) amount of accumulated glucosylceramide in the central nervous system plays a role. Increased intracerebral glucosylceramide levels have been found in Gaucher patients; a study in post-mortem human brain tissue revealed glucosylceramide levels ranging between 0.7 and 2.9 nnmol/mg in control brains, between 6.1 and 13.9 in type 1 Gaucher disease patients, between 7.8 and 15.3 in type 3 patients, and between 27.9 and 36.3 in type 2 patients (Pelled et al 2005). Neuropathologically, the hippocampal CA2-4 areas are the most consistent and characteristic regions of pathology in Gaucher disease (Wong et al 2004). This may be - partly - explained by the massive association network of these brain regions which results in a hyperexcitable state; in healthy individuals the hyperexcitable state is moderated by strong inhibitory influences, although subtle alterations of the CA2-4 neurons can result in pathologic hyperexcitability states (Wong et al 2004). Elevated glucosylceramide levels in these neurons may be such an alteration; indeed, increased glucosylceramide levels have been shown to induce an elevated intracellular calcium release in the CA2-4 neurons (Lloyd-Evans et al 2003;Korkotian et al 1999), with a significant correlation between levels of glucosylceramide and the amount of calcium release (Pelled et al 2005). Therefore, elevated intracerebral glucosylceramide levels may lead to damage to these hippocampal sub regions in patients with type 3, but also in patients with type 1 Gaucher disease, albeit to a lesser extent.

A previous study on cognitive performance in GD1 patients did not demonstrate a change in cognitive function, although slight visuo-spatial disturbances were observed (Elstein et al 2005). In our study visuo-spatial skills were not tested as such. Visuo-spatial disturbances may precede or be part of cortical and subcortical dementia syndromes. The decreased speed measures as found in our study suggest subcortical involvement rather than cortical degeneration. Indeed, diffusion tensor imaging (DTI, a tool used to investigate white matter structure) in three paediatric type 1 patients showed scattered white matter changes, although these abnormalities were largely non-specific (Davies et al 2011). Furthermore, the previously mentioned neuropathology study revealed white matter gliosis in all seven adult GD1 patients studied (Wong et al 2004).

Another interesting observation is the correlation between age and variability of attention. Although the variability of attention measure was not initially planned to be included in the analysis, it has been demonstrated to be an aspect of cognitive function specifically impaired in Dementia with Lewy Bodies (DLB) and PD dementia (PDD) when compared to patients with Alzheimer’s disease, PD or controls (Walker et al 2000; Ballard et al 2010), and was thus considered of potential interest. The hippocampal CA2-4 regions affected in type 1 as well as type 3 patients (Wong et al 2004) correspond with the neuropathological abnormalities seen in DLB which is one of the few disorders that selectively target the hippocampal CA2-3 regions. Moreover, among the Gaucher brains that were neuropathologically investigated, four patients had concomitant type 1 Gaucher disease and PDD or DLB. They all had synuclein-positive inclusions similar to the Lewy bodies seen in PD and DLB. Taking these findings together, it seems that DLB and GD1 patients have some neuropathological and cognitive features in common, albeit that the overall cognitive profile of GD1 patients is much less severe and without clinical consequence in comparison with the profile of DLB patients. These similarities are of particular interest since the two diseases have been associated with each other. One study showed that 8 out of 35 subjects with a pathological diagnosis of DLB had a mutation in the GBA gene (Goker-Alpan et al 2006).

The pathophysiological relationship between GBA and DLB has not yet been completely elucidated. It has been suggested that a (relatively) diminished enzymatic activity in Gaucher patients as well as carriers leads to an increase of glucocerebrosides in specific brain regions which can then result in neuronal dysfunction. Alternatively, mutated glucocerebrosidase (present in patients as well as carriers) might enhance aberrant fibrillization of α-synuclein and aggregation of this protein, a process that is necessary for the formation of Lewy bodies (Lwin et al 2004).

With respect to study limitations, our findings are based on a computerised system, and not on a traditional psychologist-administered neuropsychological test battery. However, the CDR system has been proven to be a valid measure of cognitive function (Simpson et al 1991). Moreover, advantages of the CDR system are the objective nature of the tests, which can be administered in multiple languages and the short time needed to complete the test battery (30 minutes). The age-matched normative sample is large which makes comparisons between diseased and healthy people valuable. In contrast to these healthy controls, however, GD1 patients underwent the cognitive assessment among other investigations which might have led to an underestimation of their cognitive capacities due to fatigue or situational anxiety. However, the above average performance on the composite scores continuity of attention, quality of working memory and quality of episodic memory makes it unlikely that these factors played a major role in the findings of the present study. Besides, our patients were compared to healthy controls. We do not know to what extent the impaired speed measures found in the present study are due to suffering from a chronic disease in general rather than GD1 itself. However, patients with for instance rheumatoid arthritis showed a normal cognitive profile, making it unlikely that having a chronic disease is the main cause of our findings (Park et al 1999). The absence of a main effect of time-point in this study is probably due to the relatively short follow-up time. The decline in power of attention from baseline without progressive decline thereafter may have been due to the absence of training sessions prior to the follow-up visits. The pattern of improvement of quality of episodic memory and speed of memory may have reflected a training effect with repeated assessments. However, such effects have not been seen in other studies with healthy controls (Wesnes et al 2000).

Despite these shortcomings, we feel confident that findings from this study are robust since GD1 patients with concomitant diseases that could distort our results were all excluded. We therefore propose that our observations are due to GD1 itself.

## Conclusion

GD1 patients develop impaired cognitive speed measures, although the impact on everyday life was not assessed. Further studies are needed to establish whether these abnormalities are related to Gaucher disease itself.
